# Optimization of Intra-Arterial Administration of Chemotherapeutic Agents for Glioblastoma in the F98-Fischer Glioma-Bearing Rat Model

**DOI:** 10.3390/biom15030421

**Published:** 2025-03-16

**Authors:** Juliette Latulippe, Laurent-Olivier Roy, Fernand Gobeil, David Fortin

**Affiliations:** 1 Department of Pharmacology and Physiology, Faculty of Medicine and Health Sciences, Université de Sherbrooke, Sherbrooke, QC J1H 5N4, Canada; juliette.latulippe@usherbrooke.ca (J.L.); fernand.gobeil@usherbrooke.ca (F.G.); 2 Division of Neurosurgery, Department of Surgery, Centre Hospitalier de l’Université de Sherbrooke, Sherbrooke, QC J1H 5N4, Canada; laurent-olivier.roy@usherbrooke.ca

**Keywords:** chemotherapeutic agents, topotecan, cytarabine, carboplatin, paclitaxel, glioblastoma, blood–brain barrier

## Abstract

Glioblastoma (GBM) is a difficult disease to treat for different reasons, with the blood–brain barrier (BBB) preventing therapeutic drugs from reaching the tumor being one major hurdle. The median overall survival is only 14.6 months after the standard first line of treatment. At relapse, there is no recognized standard second-line treatment. Our team uses intra-arterial (IA) chemotherapy as a means to bypass the BBB, hence achieving an overall median survival of 25 months. However, most patients eventually fail the treatment and progress. This is why we wish to expand our portfolio of options in terms of chemotherapy agents available for IA administration. In this study, we tested topotecan, cytarabine, and new formulations of carboplatin and paclitaxel by IA administration in the F98-Fischer glioma-bearing rat model as a screening tool for identifying potential candidate drugs. The topotecan IA group showed increased survival compared to the intravenous (IV) group (29.0 vs. 23.5), whereas the IV cytarabine group survived longer than the IA group (26.5 vs. 22.5). The new formulation of carboplatin showed a significant increase in survival compared to two previous studies with the conventional form (37.5 vs. 26.0 and 30.0). As for paclitaxel, it was too neurotoxic for IA administration. Topotecan and the new formulation of carboplatin demonstrated significant results, warranting their transition for consideration in clinical trials.

## 1. Introduction

Glioblastoma (GBM) is the most aggressive primary malignant brain tumor in adults. The overall survival is only 14.6 months, and the progression-free survival is 6.9 months after the first line of treatment, which consists of maximal surgical resection followed by concomitant radiotherapy and chemotherapy with temozolomide [1]. There is no consensus on the optimal therapeutic method to use at relapse. Hence, every clinical team offers treatment at relapse based on their own experience, biases, and resources. GBM remains challenging to treat due to the limited penetration of therapeutic drugs in the brain parenchyma, primarily restricted by the blood–brain barrier (BBB). Indeed, the BBB, a selectively impermeable barrier formed by the microvasculature of the central nervous system (CNS), safeguards the healthy brain by blocking most blood-borne substances but also hinders effective treatments of CNS diseases [2,3].

Consequently, most chemotherapeutic agents (CTAs) administered intravenously (IV) or orally fail to cross the BBB, while others achieve only partial penetration, preventing the accumulation of therapeutically effective concentrations in tumor cells [4]. To overcome this limitation, various strategies, such as regional or local therapies, have been proposed to bypass the BBB and target the bulk of the tumor more effectively [5]. At the Centre Hospitalier de l’Université de Sherbrooke (CHUS), our team elected to deliver the CTAs intra-arterially (IA) to increase the accumulation of therapeutic drugs in the diseased brain. IA delivery offers benefits that are not found in traditional IV or oral administrations, such as selective infusion, an increase in drug concentration via the first pass effect, and also offering the option of manipulating the BBB permeability via different means [6,7]. Clinical studies conducted by our team on GBM patients (n = 319) have demonstrated that IA administration has the potential to extend the overall median survival to 25 months [8], which is quite significant compared to the current standard of care first-line treatment (overall median survival: 14.6 months) [1]. Moreover, IA drug delivery is a safe procedure and decreases systemic adverse effects as the entire administration is delivered to the brain tumor area, comparatively to IV infusions [9,10,11]. This decreases the systemic recirculation of the drug by up to 20% [12]. Currently, only five CTAs are used in the clinic by our team, as each agent needs to be carefully selected and tested for efficacy as well as safety in IA administration. We intend to improve our drug portfolio for IA administration to increase options for our patients [8]. This will eventually allow us to use drug sensitivity testing to construct personalized drug protocol solutions for each patient in the hope of decreasing drug resistance and improving outcomes.

This study compares four different CTAs (topotecan, cytarabine, carboplatin, and paclitaxel) in two different administration routes (IA and IV). Topotecan is a topoisomerase I inhibitor that has demonstrated a beneficial effect on proliferating glioma cells [13]. It can also partially penetrate the intact BBB when administered IV [14]. Cytarabine is an S-phase-specific antimetabolite, classified as a pyrimidine analog, that is commonly used to treat CNS disorders, such as primary CNS lymphoma [15]. Carboplatin is a platinum alkylating agent currently used to treat CNS tumors in IA administration by our team, with or without BBB opening [16,17]. Considering that this agent has already shown a response in IA administration, we included a new formulation of carboplatin in this study. The carboplatin used was solubilized in a new confidential vehicle specifically developed and produced by the company Ingenew BioPharma. Finally, paclitaxel is a microtubule-stabilizing CTA that represents a great advance in oncology, with clinical responses in a variety of primary cancers. Unfortunately, this drug presents a poor penetration across the BBB when infused IV, but localized therapy with nanoparticles has shown promising results [18,19,20]. Paclitaxel has previously been tested by IA with Cremophor as a solubilization vehicle, but the animals did not survive the procedure as the vehicle itself seemed to produce fatal side effects (data not published). Hence, the use of the new confidential formulation by Ingenew BioPharma, incorporating an alternative solubilizing agent, was evaluated to determine whether IA administration would be feasible and beneficial.

The principal aims of this study were to (1) determine the maximal tolerable dose (MTD) and the innocuity of the previously mentioned CTAs administered IA in a healthy rat model and (2) evaluate their therapeutic effects in a glioma-bearing rat model.

## 2. Materials and Methods

### 2.1. Chemicals

Topotecan (4 mg/mL) and cytarabine (100 mg/mL) were purchased from the oncology pharmacy of the CHUS (Sherbrooke, Québec, Canada). Paclitaxel (25 mg/mL) and carboplatin (20 mg/mL) were provided by the pharmaceutical industry Ingenew BioPharma (Laval, Québec, Canada).

### 2.2. Cell Line and Culture Conditions

The cell line chosen for this project was the F98 glioma cells (American type culture collection—Manassas, VA, USA; ATCC CRL-2397), as this cell line is syngeneic with the Fischer rat. Thereby, we avoid variations that could arise from immune responses produced in non-immunosuppressed animals by other cell lines. The F98-Fischer model was also chosen since it recreates GBM’s behavior of humans well. Maintenance and preparation for our experiments were as previously described [21].

### 2.3. Animals

For the evaluation of the MTD for each chemotherapeutic agent, male Wistar rats were used. These animals were not implanted with glioma cells, as the goal was to assess toxicity at different dose levels. For the survival efficacy study, male Fischer rats were used. All rats were purchased at Charles River Laboratories (Saint-Constant, Québec, Canada). For every procedure (implantation, chemotherapy infusion, euthanasia), animals were anesthetized with isoflurane (2–2.5%, O_2_ at 2 L/min). Every experimental protocol conformed to the regulations set by the Canadian Council on Animal Care and was approved by the institutional ethical committee of the Université de Sherbrooke (protocols ID: 2022-2856 and 2023-3922).

### 2.4. Drugs Administration

**Figure 1:**
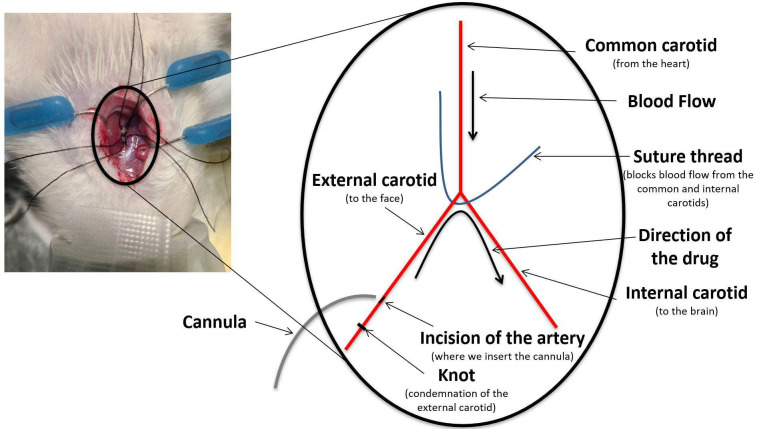
Schematic representation of the IA surgery.

The IA procedure involved drug delivery through a catheter inserted retrogradely into the right external carotid artery using PE-10 intramedic tubing, with the catheter tip positioned right above the bifurcation. This setup allowed for retrograde infusion into the external carotid artery, transitioning to orthograde flow into the internal carotid artery, ultimately directing the drug into the right hemisphere of the brain. After the infusion, the external carotid was condemned, and the animal was closed with sutures (Figure 1). This procedure was performed as previously described by Fortin et al. [22,23]. The IV infusions of CTAs were administered via the caudal tail vein. The intra-tumoral (IT) injections of CTAs were performed over the three-minute duration using the same coordinates as those employed for the implantation procedure (see below). Since the MTD study was conducted on tumor-free animals, the IA infusion was the first procedure performed. In contrast, for the survival study, animals received the treatment 10 days following the F98 glioma cell implantation.

### 2.5. Treatment Groups 

**Figure 2:**
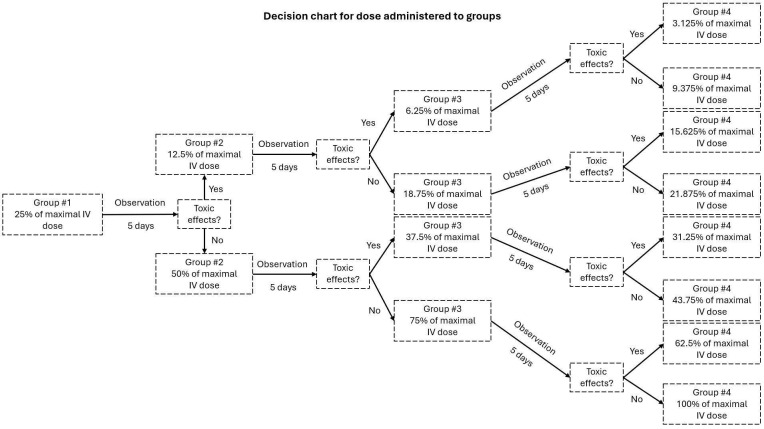
Graphic illustrating the decision chart representing the administered dose for each group.

For the MTD study, there were four independent groups with three Wistar rats each for every CTA tested. Each group corresponded to a different dose (Figure 2). As for the efficacy study, a total of eight groups were planned, with four animals in each group. However, some animals were added to different groups to increase the sample size either to compensate for the loss of some animals because of events unrelated to the tumor or to validate drug efficacy (topotecan and carboplatin) or lack thereof (cytarabine). The final number of animals per group was as follows: (1) Topotecan IA (n = 5), (2) topotecan IV (n = 4), (3) cytarabine IA (n = 6), (4) cytarabine IV (n = 4), (5) control IA (n = 4), (6) carboplatin IA (n = 8), (7) paclitaxel IT (n = 4), and (8) control IT (n = 4). Note that six animals were used for IA administration of paclitaxel at different doses, while two received only the vehicle; however, the route of administration had to be changed due to toxicity.

### 2.6. MTD Evaluation

The starting dose for the first group was 25% of the maximal IV dose found in the literature [24,25]. After five days of observation, the animal was euthanized, and the brain, the heart, a kidney, parts of the spleen, the lungs, and the liver were harvested for histology. A decision scheme was used to select the subsequent doses (Figure 2). Briefly, if there was any sign of toxicity, behaviorally or in the organs, the second group received half the dose of the previous one (12.5% in this case). If no sign of toxicity was detected, the dose was doubled (50%). This paradigm was followed until the fourth group was reached.

### 2.7. Implantation

This procedure was only performed for the efficacy study using the F98-Fischer rat model. Confluent F98 cells were harvested and resuspended in non-supplemented warm Dulbecco’s Modified Eagle medium (DMEM) at a concentration of 2000 cells/µL. This procedure was performed as described by Blanchard et al. [21]. Briefly, 10,000 cells (5 µL) were implanted in the right frontal lobe, 3 mm laterally and 1 mm anteriorly from the bregma at a depth of 5 mm.

### 2.8. Evaluation of Mean Survival Time

Animals were monitored daily. Loss of self-grooming (periocular secretion accumulation), landing ability, coordination, stability, mobility, and weight measurement were performed. The presence of convulsions was also monitored. The animals were euthanized if they reached a score of 3/10 or less in 3 categories or if they lost more than 20% of their initial weight. Euthanasia was performed under anesthesia with isoflurane (2–2.5%, O_2_ at 2 L/min) via a 60 mL intracardiac perfusion of 4% paraformaldehyde (PFA). The brain was harvested and conserved in 4% PFA before being transferred in 70% ethanol 24 h later and maintained at 4 °C until preparation for histology.

### 2.9. Histology

Haemotoxylin and eosin (H&E) staining was performed by the Université de Sherbrooke’s histology platform on all harvested specimens. For histological examination, tissue samples were preserved in 10% buffered formalin, embedded in paraffin, and sectioned into 4 μm-thick slices, which were then stained with hematoxylin and eosin (H&E). High-resolution images of the H&E-stained sections were obtained using a Nanozoomer 2-slide scanner (Olympus) and analyzed with the NDP view 2 imagingsoftware (version 2.6.13) [26]. The H&E staining was used to determine if there was any sign of toxicity in the MTD study and to identify the necrotic and tumoral areas in the survival study.

### 2.10. Statistical Analysis

Data for the survival study were analyzed by the Kaplan–Meier survival curves using a Log-Rank test. *P*-values under 0.05 were considered statistically significant.

## 3. Results

### 3.1. MTD Study

Since carboplatin’s IA dosage had already been determined by our group, only topotecan, cytarabine, and paclitaxel were tested in this study [27].

**Figure 3:**
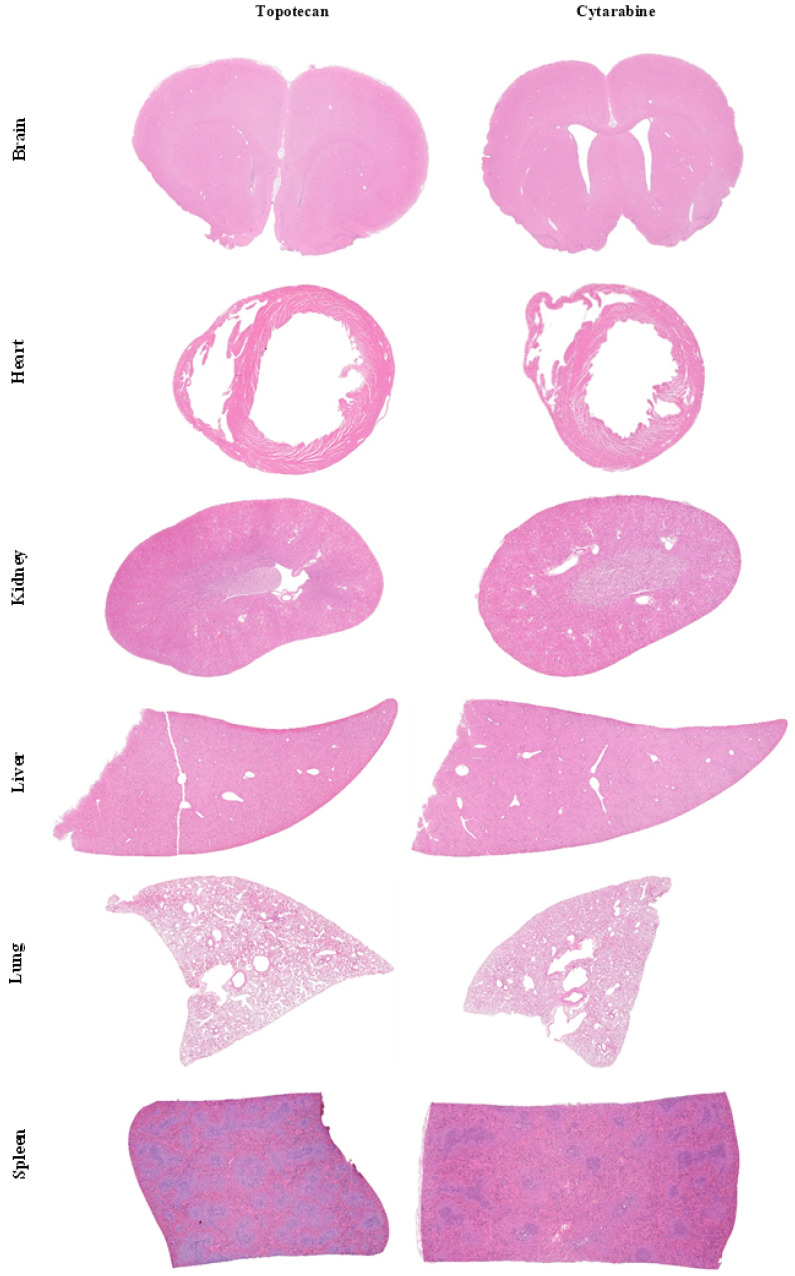
H&E images of vital organs at the end of treatments showing the non-toxicity of topotecan and cytarabine when administered IA at 4 mg/kg and 8.75 mg/kg, respectively, which were their highest doses tested. Magnification: 20×.

Topotecan was obtained from a 1 mg/mL solution. The IV dose of 4 mg/kg was selected based on the literature [24]. As per the dose decision scheme (Figure 2), the first IA group received 1 mg/kg (Table 1). There was no treatment-related complication nor sign of toxicity in the organs in the first group. The other three groups showed a similar outcome. No abnormal signs in the animals’ behavior were observed. Likewise, no toxicity was observed in the brain, heart, kidney, spleen, lung, or liver based on the H&E sections (Figure 3). Therefore, following the dose escalation scheme described above, the selected treatment dose for IA infusion was 4 mg/kg, representing 100% of the IV dose.

Cytarabine’s first tested dose was 10 mg/kg from a 100 mg/mL solution, as the IV dose was 40 mg/kg (Table 1) [25]. This dosage was considered too toxic as the animals did not survive the entire procedure. Indeed, they presented heavy tachycardia and died immediately or soon after the end of the IA infusion. The subsequent dose was, thus, reduced by half. The second, third, and fourth groups tolerated their dosages well, and there were no apparent signs of toxicity (Figure 3). The IA cytarabine MTD was, therefore, 8.75 mg/kg, which represented almost 22% of the maximal IV dose.

As for paclitaxel, no animals survived the IA infusions. During the first administration (3.3 mg/kg), animals displayed abnormally irregular and jerky breathing (Table 1). The second and third doses (1.65 and 0.825 mg/kg) also resulted in mortality of the animals during the infusions. We repeated this procedure using only the vehicle at a volume matching the last tested dosage to verify whether this effect was caused by the CTA or not. Unfortunately, the animals also died during the administration. From that point on, IA administration was substituted for IT infusions for survival studies using paclitaxel.

### 3.2. Efficacy/Survival Study

**Figure 4:**
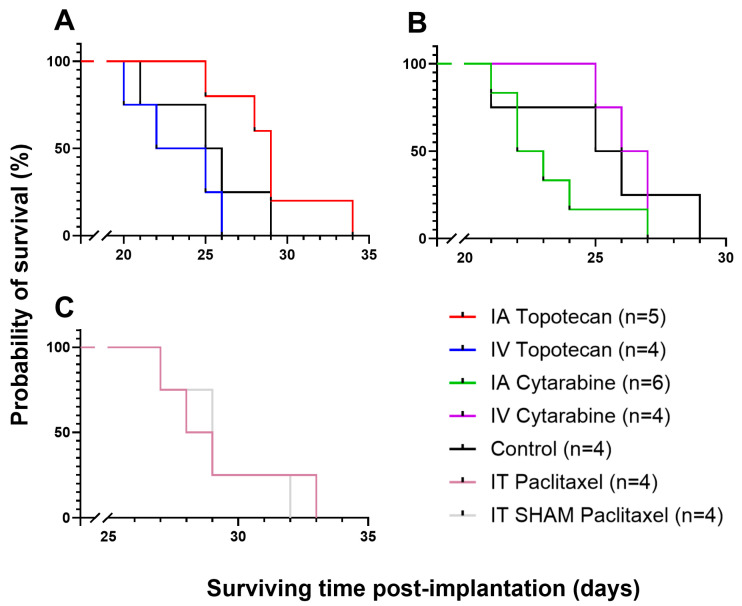
Kaplan–Meier survival analysis of F98 glioma-bearing rats following treatment. (**A**) Topotecan by IA and IV and its control. (**B**) Cytarabine by IA and IV and its control. (**C**) IT paclitaxel and SHAM.

To evaluate the efficacy of topotecan and cytarabine using IA infusion at selected doses (Table 1), we conducted survival studies comparing these groups’ survival with IV and control groups. Topotecan showed a significant increase in survival for the IA group, with a median of 29.0 days compared to 23.5 days for the IV group (*p* = 0.015). However, there was no significant difference between the IA group and the control group (Figure 4, Table 2). The control group’s median survival (25.5 days) is consistent with our previously reported standardization experiments, which described a median survival of 26 ± 2 days for untreated animals [28].

The group receiving IA cytarabine showed a lower survival rate with a median of 22.5 days compared to the IV and control groups at 26.5 and 25.5 days, respectively (Figure 4, Table 2). These results demonstrate that IA administration appears more toxic than IV delivery, suggesting that this approach is not advisable for clinical use.

Carboplatin was used at a dose previously described by our group (20 mg/kg) from 20 mg/mL [27,29]. This new solution showed interesting results, as the median survival of the IA group was 37.5 days (n = 8). This CTA was not compared with an IV group since the goal of this study was to determine if this new formulation was more efficient than the carboplatin diluted in saline (median survival of 26.0 days, n = 8) or 5% dextrose (median survival of 30.0 days, n = 9). Table 3 illustrates the results of this study alongside two others conducted by our group using the same animal model and the other two vehicles [27,29]. The newly tested carboplatin formulation demonstrated improved efficacy in enhancing the survival of the animals.

Finally, since paclitaxel could not be administered IA, we tested its effect when administered via the IT route (375 µg/kg from a 25 mg/mL solution [30]). A SHAM group, in which animals received an IT infusion of the vehicle alone, was included. The group treated with paclitaxel achieved a median survival of 28.5 days, while the SHAM group survived a median of 29.0 days, showing no statistically significant difference in survival outcomes (Figure 3, Table 2).

## 4. Discussion

GBM patients still present a short median survival despite decades of research. One of the main reasons for this dreadful outcome is the selective impermeability of the BBB, preventing a significant buildup of therapeutic concentration in the CNS. Even though IA infusions of CTAs increase survival, most patients who respond eventually recur and progress while developing chemoresistance [8]. Since IA chemotherapy has proven effective as a treatment at first relapse, we aim to expand the range of available CTAs that can be administered via this route.

Hence, to improve our therapeutic portfolio of available CTAs, four distinct drugs were selected and tested for IA perfusion in the F98-Fischer preclinical syngeneic model of GBM. These agents were chosen as they either were already used to treat brain tumors IV, cross partly the BBB, show good potential to treat aggressive tumors, or were presented under a new formulation that could possibly lead to better results than those already used in clinical care [8,14,15,18].

In the treatment of GBMs, the administration routes are classically the oral or IV route. However, these delivery strategies administer the CTAs systemically, thus decreasing their accumulation in the brain and the tumor. Indeed, the survival results of our IV studies using topotecan and cytarabine are consistent with those obtained in the control group, suggesting that they can barely, if at all, cross the BBB (Figure 4). On the other hand, Charest et al. showed that platinum compounds administered IA would accumulate significantly more in the tumor compared to IV infusions [27]. IA drug delivery has also been shown to be safe and efficacious in brain tumors in the clinical setting when the CTAs were adequately selected [10,11,31,32]. It appears to be as safe as diagnostic cerebral angiography, as the complications arise mostly from the catheter placement and are extremely unusual (0.75%) [11]. This route has also been utilized in children and was well tolerated [33]. Additionally, CTAs are not the only compounds that can be administered IA. Many clinical trials have used monoclonal antibodies for different types of brain diseases [31,33]. Furthermore, BBB disruption (BBBD) prior to IA infusions is a well-established procedure that can increase up to 300 times the local concentration of CTAs in the brain compared to IV infusion [7]. Hence, BBBD serves as a valuable adjunct to IA infusion. Although BBBD was not utilized in this study, it is a strategy worth considering for future investigations.

Considering that topotecan, cytarabine, and paclitaxel have never been tested IA, the first step was to determine their maximal tolerable dose with no observable adverse effects. Our results showed that neither topotecan nor cytarabine produced neurotoxicity when tested at 4 mg/kg and 8.75 mg/kg, their respective highest tolerated dose. The same could be said of systemic innocuity, as the heart, the kidneys, the liver, the lungs, and the spleen were also surveyed to confirm the absence of toxic effects of these drugs. Our results suggest that these drugs can be safely administered IA at these doses without causing any adverse effects. However, none of the animals survived the IA administration of paclitaxel. Preliminary experiments revealed that the vehicle was responsible for the observed neurotoxicity when infused via the IA route, prompting a switch to IT infusions for our survival studies.

The IA topotecan group showed an increase in survival compared to the IV group. Indeed, while the median survival of the IV group was not significantly different from the controls, the IA group presented a median survival significantly higher with 29 days (*p* = 0.015). Hence, considering that this drug seems to be well tolerated by the animals and produced an increase in survival, it would be interesting to do a Phase 0–1 clinical trial (escalating dose and innocuity assessment) to determine its usefulness in our population of relapsing GBM patients. This is not the first attempt to use this drug with a regional delivery strategy. In fact, other research groups have used convention-enhanced delivery (CED) of topotecan in both small and large animals and reported great results with significantly higher survival using this method [34]. Our results align with theirs while using a single IA infusion of the therapeutic agent. Moreover, Spinazzi et al. complemented these previous findings in a clinical study where they used the same agent and method. While further studies would be needed to prove favorable outcomes, their small group showed that CED is a potentially safe and effective treatment for recurrent GBM patients [13]. Hence, topotecan could be a good option for regional therapy via IA administration in relapsing GBM patients.

In contrast, the cytarabine groups revealed that the IV-treated rats survived longer than those treated via IA administration, although the difference was not statistically significant (*p* = 0.0523). Additionally, the IV group did not exhibit any significant differences in survival compared to the control group. This suggests that cytarabine is ineffective despite the treatment being well tolerated by the animals. It is also important to note that the control group used with topotecan and cytarabine displayed irregularities. More specifically, four animals were used in this group and had survival times of 21, 25, 26, and 29 days, respectively, resulting in a median survival of 25.5 days. While this is technically within the range reported in the literature (26 ± 2 days), such variation has never been observed previously in our lab [28].

The new IA carboplatin formulation (Ingenew BioPharma) showed improved survival compared to the standard formulation (Novopharm) used in our laboratory in preclinical and clinical studies, with a median survival of 37.5 days. In the past, certain diverging results obtained by our laboratory hinted at the fact that the solubilization vehicle might influence results. Hence, using the same F98-Fischer glioma-bearing rat model, Côté et al. described a median survival of 26 days with carboplatin in saline, while Charest et al. reported a median survival of 30 days when 5% dextrose was used as a vehicle [27,29]. The new carboplatin formulation, submitted for patent by Ingenew BioPharma, appears to surpass these results and could potentially replace the formulation currently used in our patients in the clinic, pending confirmation in a clinical comparative study.

The standard vehicle for paclitaxel is Cremophor, as the drug is not soluble in saline. However, Cremophor itself is a toxic compound when delivered via the IA route. Indeed, our lab has previously attempted to use the IA route for the delivery of paclitaxel with its usual vehicle, but severe instances of neurotoxicity occurred and resulted in fatal outcomes. In fact, Cremophore caused severe convulsions during the infusion (data not published). Hence, the IA infusion of placlitaxel with this compound was promptly interrupted. In this study, paclitaxel was dissolved using a new formulation developed by Ingenew BioPharma. Since Cremophor was identified as the cause of the toxic effect observed previously, testing this new formulation for IA delivery was of interest. Unfortunately, the new vehicle was also poorly tolerated by the animals, as none survived the treatment. IT administration of paclitaxel was, thus, instead implemented to see if better results could be obtained, but it did not show a significantly higher survival compared to the SHAM group (28.5 vs. 29 days). The results from the SHAM group were unexpected, as these animals received only the vehicle and were, therefore, expected to exhibit the same median survival as the control group. Interestingly, all animals that received either the drug or the vehicle via the IT route showed brain hemorrhage. This could, of course, be attributed to the needle used for the procedure (23G); however, the same needle size is routinely used for the implantation procedure without any adverse events. The administration of paclitaxel was performed on day 10 post-tumor cell implantation. Given that tumors are more highly vascularized compared to healthy brain parenchyma, it is possible that the needle might have damaged blood vessels in the tumor, leading to hemorrhages.

In conclusion, two of the four drugs tested in this study showed an increase in median survival rates, while the other two were either too toxic or unsuitable for IA infusion in the F98-Fischer glioma-bearing rat model. BBBD could be considered in future studies to further enhance these efficacy results. Phase 0–1 clinical trials will be planned to evaluate the new Ingenew BioPharma carboplatin formulation and topotecan.

## Figures and Tables

**Table 1:** First and final dosage used in the MTD and survival study.

CTA	1st Dosage	4th Dosage	% of the IV Dosage	Dosage Used for Survival Studies
Topotecan	1 mg/kg	4 mg/kg	100%	4 mg/kg
Cytarabine	10 mg/kg	8.75 mg/kg	21.88%	8.75 mg/kg
Paclitaxel	3.3 mg/kg	0.825 mg/kg	-	Too toxic

**Table 2:** Median survival times of the different groups.

Chemotherapy Agent and Administration Route	Median Survival (Days)
Topotecan—IA	29.0
Topotecan—IV	23.5
Cytarabine—IA	22.5
Cytarabine—IV	26.5
Control	25.5
Carboplatin (Ingenew‘s formulation)—IA	37.5
Paclitaxel (Ingenew’s formulation)—IT	28.5
Paclitaxel (Ingenew’s formulation) SHAM—IT	29.0

**Table 3:** Median survival times of animals treated with carboplatin in three different studies.

Study	Côté et al. (2013) [29]	Charest et al. (2013) [27]	Present Study
Drug	Carboplatin (saline)	Carboplatin (5% dextrose)	Carboplatin (Ingenew’s formulation)
Median survival (days)	26.0 ***	30.0 **	37.5

** *p* < 0.01, *** *p* < 0.001 vs. new carboplatin formulation from Ingenew BioPharma, based on Log-Rank test.

## Data Availability

The data used and/or analyzed in the current study are available from the corresponding author upon reasonable request.

## Abbreviations

The following abbreviations are used in this manuscript:

BBB	Blood–brain barrier
CED	Convection-enhanced delivery
CHUS	Centre Hospitalier de l’Université de Sherbrooke
CNS	Central nervous system
CTA	Chemotherapy agent
DMEM	Dulbecco’s modified eagle medium
GBM	Glioblastoma multiform
H&E	Hematoxylin and eosin
IA	Intra-arterial/intra-arterially
IT	Intratumoral
IV	Intravenous
MTD	Maximal tolerable dose
BBBD	Blood–brain barrier disruption
PFA	Paraformaldehyde
